# Targeting KRAS in Lung Cancer Beyond KRAS G12C Inhibitors: The Immune Regulatory Role of *KRAS* and Novel Therapeutic Strategies

**DOI:** 10.3389/fonc.2021.793121

**Published:** 2022-01-13

**Authors:** Marc Cucurull, Lucia Notario, Montse Sanchez-Cespedes, Cinta Hierro, Anna Estival, Enric Carcereny, Maria Saigí

**Affiliations:** ^1^ Department of Medical Oncology, Catalan Institute of Oncology (ICO), Barcelona, Spain; ^2^ Badalona·Applied Research Group in Oncology (B·ARGO), Institut d’Investigació en Ciències de la Salut Germans Trias i Pujol (IGTP), Barcelona, Spain; ^3^ Cancer Genetics Group, Josep Carreras Leukemia Research Institute (IJC), Barcelona, Spain

**Keywords:** KRAS, NSCLC, ICI, PD-L1, STK11

## Abstract

Approximately 20% of lung adenocarcinomas harbor *KRAS* mutations, an oncogene that drives tumorigenesis and has the ability to alter the immune system and the tumor immune microenvironment. While KRAS was considered “undruggable” for decades, specific KRAS G12C covalent inhibitors have recently emerged, although their promising results are limited to a subset of patients. Several other drugs targeting KRAS activation and downstream signaling pathways are currently under investigation in early-phase clinical trials. In addition, *KRAS* mutations can co-exist with other mutations in significant genes in cancer (e.g., *STK11* and *KEAP1*) which induces tumor heterogeneity and promotes different responses to therapies. This review describes the molecular characterization of *KRAS* mutant lung cancers from a biologic perspective to its clinical implications. We aim to summarize the tumor heterogeneity of *KRAS* mutant lung cancers and its immune-regulatory role, to report the efficacy achieved with current immunotherapies, and to overview the therapeutic approaches targeting *KRAS* mutations besides KRAS G12C inhibitors.

## Introduction

After decades of research, the treatment efficacy of advanced lung cancer has remarkably improved, by incorporating novel therapeutic strategies including targeted therapies inhibiting specific genetically activated proteins, or immunotherapies such as immune-checkpoint inhibitors (ICI) (1). Mutations affecting members of the RAS family genes (*KRAS, HRAS, NRAS*) are the most frequent genetic alterations in human cancer, affecting about 27% of all tumors, including lung, colorectal and pancreatic ductal adenocarcinoma, among others (2, 3).

**Figure 1:**
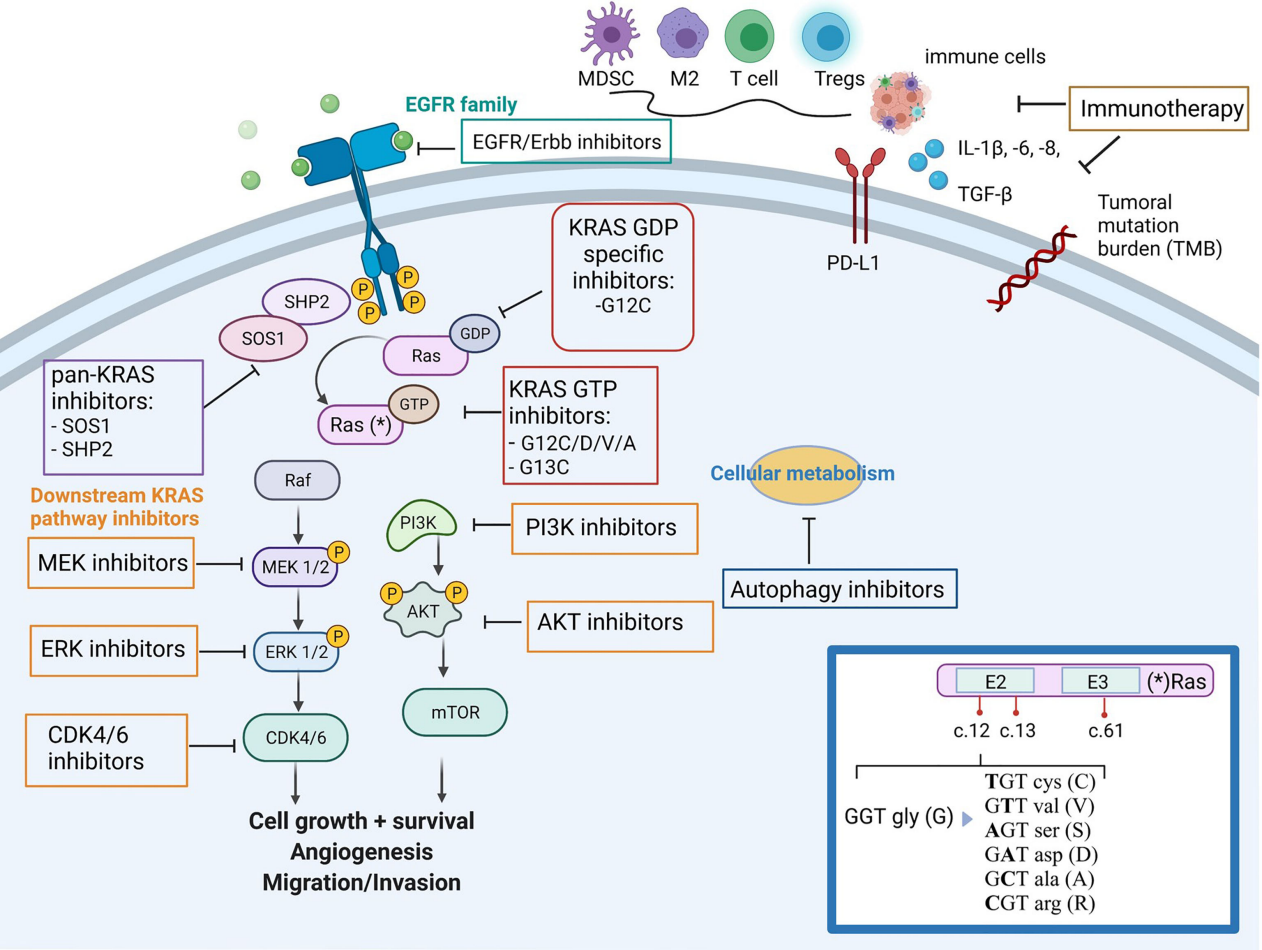
Simplified overview of *KRAS* mutant tumor, downstream/upstream KRAS pathways, its impact on the immune-microenvironment and family of drugs targeting *KRAS* mutant tumors. (*) In the inset box, the oncogenic mutations in codon 12 of *KRAS* and aminonacid changes. *Figure has been created with BioRender.com*.

Lung adenocarcinoma (LuAD) is a type of cancer with the largest number of oncogenic alterations that are therapeutically approachable (4). Approximately 20-25% of the LuADs harbor *KRAS* mutations, most of them affecting codons 12 (~85%), 13 (~10%) or 61 (~5%). In LuADs from smokers, the vast majority of *KRAS* mutations consist on guanine to thymine transversions, an effect that is associated with the tobacco carcinogens (5). At the aminoacid level, most of these mutations replace the glycine (G) in codon 12 by a cysteine (C) (G12C) and occurs in almost 50% of *KRAS* mutant tumors. On the other hand, *KRAS* mutations in never-smokers are less frequent, and the most prevalent changes involve nucleotide transitions that replace the glycine in codon 12 by an aspartic acid (G12D) or a valine (G12V) (6, 7) (**Figure 1**).

In contrast to other oncogenic proteins activated in cancer and despite multiple attempts to harness it, KRAS was considered undruggable for a long time. The KRAS mutated proteins has a reduced capability to hydrolyze GTP or to interact with the GTPase-activating proteins, maintaining the oncogene and the downstream pathways constitutively activated. The lack of specific inhibitors targeting the KRAS hydrophobic pocket and the complexity of downstream pathways have contributed to the challenge of developing effective therapeutic strategies (8). After years of study, novel KRAS selective inhibitors became available for the KRAS G12C mutant protein, enabling a covalent binding that hinders downstream signaling, which led to promising results in the clinical setting. Two specific KRAS G12C inhibitors, first sotorasib (AMG510) and later adagrasib (MRTX849), earned the breakthrough designation by the US Food and Drugs Administration (FDA), to treat metastatic lung cancer patients harboring that particular *KRAS* mutation who have progressed to at least one prior systemic therapy. In the case of sotorasib, this was based on the efficacy results of the phase I/II CodeBreak-100 trial (NCT03600883) that reported an objective response rate (ORR) of 32% and disease control rate (DCR) of 88% among lung cancer patients. Thus, sotorasib has the FDA-approval for this clinical indication (9). On the other hand, data from the phase I/II KRYSTAL-1 trial (NCT03785249) for adagrasib showed significant benefit with an ORR of 45%, although the study is still ongoing and definitive conclusions cannot be inferred (10). Many other drugs targeting KRAS activation and other parallel and downstream pathways, as well as immunotherapeutic strategies, are currently under investigation in early-phase clinical trials (11).

Here, we aim to describe the tumor heterogeneity of *KRAS* mutant lung cancers and its immune-regulatory role, to report the efficacy with current immunotherapies, and to overview the therapeutic approaches targeting *KRAS* mutant tumors, other than KRAS G12C inhibitors.

## *KRAS-*Mutant Lung Cancer Is a Heterogeneous Disease and Has a Characteristic Immune-Microenvironment

Mutations on *KRAS* and on other actionable oncogenic drivers, such as *EGFR* or *ALK*, are often mutually exclusive. However, they co-occur with mutations in important tumor suppressor genes, such as *STK11* (also known as *LKB1), KEAP1*, *TP53*, or *CDKN2A*, whose inactivation cooperates with *KRAS* in the oncogenic process and, thus, characterize the heterogeneous nature of *KRAS* mutant tumors (12, 13). In addition, the different KRAS mutated proteins differ on their biologic properties to hydrolyze GTP and to activate downstream signaling pathways, which determines the differences in their therapeutic vulnerabilities (14, 15).

Skoulidis et al. described a molecular classification of *KRAS* mutant LuAD according to the presence of co-mutations in tumor suppressor genes *TP53 (~30%)* and/or LKB1 (~30% each) (16). These co-mutation partners lead to variances in gene expression and distinct patterns of inflammatory and immune-checkpoint molecules release, which models the tumor microenvironment and promotes different responses to therapies (12, 17). Because of this tumor heterogeneity, the prognostic role of *KRAS* mutant cancers remains uncertain, although most studies report a major aggressive behavior of this type of cancer (18–20).

*KRAS* mutant tumors are characterized by enabling tumor cells to escape immunosurveillance as one of the hallmarks of cancer (21). NF-κB, STAT3, and certain suppressive inflammatory cytokines such as IL-6, IL-1β and GM-CSF, are key transducers of the immunosuppressive properties of *KRAS* driven tumors (22–24). Other mechanisms in this tumor type consist in increasing the expression of immune-checkpoints (e.g., PD-L1) to prevent T-cell effector functions, eliciting the release of myeloid-derived suppressor cells, regulatory T cells, and M2-differentiated tumor-associated macrophages, all of which impair antitumor immunity and facilitates tumor growth (25, 26) (**Figure 1**).

In preclinical mice models, *Stk11/Kras* mutant tumors produced abundant IL-6 and were associated with neutrophil accumulation and inflammatory cytokines with immunosuppressive properties in the tumor microenvironment, along with increased levels of T-cell exhaustion markers, compared with normal lungs (27). Accordingly, *KRAS/STK11*-mutant tumors are associated to a “cold immunophenotype” with lower T-cell infiltration and lower rates of PD-L1 immunostaining, among other immunosuppressive features. Lung cancer patients with this tumor genetic profile showed a worse response to ICI than did patients with *KRAS-*mutant tumors without *LKB1* co-mutations (27–30).

More recently, *STK11* inactivation in *KRAS-*mutant tumors, has been shown to enhance the silencing of the STimulator of INterferon Genes (STING) protein, in part through epigenetic mechanisms (31). STING is a key component of the innate immunity and acts as a sensor for double-strand DNA (dsDNA) in the cytosol from virus and pathogens, which mediates the type I interferon production (32). By silencing STING expression, *KRAS/STK11-*mutant tumors become insensitive to cytoplasmatic dsDNA sensing, avoiding T-cell inflammation and promoting the recruitment of exhausted T-cell lymphocytes. Thus, restoring STING expression or activation of the cyclic GMP–AMP synthase (cGAS)-STING pathway with STING agonists, could induce T cell infiltration and turn this subset of tumors into more immunogenic, increasing the synergistic effects in combination with ICI (33).

## The Efficacy of Current Immunotherapies in Lung Cancers With *KRAS* Mutations

Besides the immune-related nature of *KRAS* driven tumors, the smoking habit of patients with *KRAS* mutations has been associated with higher tumor mutational burden (TMB), which might predict better responses to ICI (34, 35).

Mazieres et al. retrospectively evaluated a cohort of non-small cell lung cancer (NSCLC) harboring different oncogenic driver mutations. Among them, the subset of *KRAS* mutant tumors expressed higher rates of PD-L1 and responded better to ICI than tumors with other oncogenic driver alterations (36). On the other hand, most phase 3 clinical trials evaluating all-comers with NSCLC treated with ICI did not stratify by *KRAS* status, and only *post-hoc* analyses have been performed on that subset. The following section compiles the evidence derived from exploratory analyses of the most relevant phase 3 clinical trials evaluating ICI and real-world data from retrospective cohorts of NSCLC patients treated with ICI.

The efficacy of nivolumab has been reported in two international studies. In the Italian Expanded Access Program out of the 530 patients who received 2^nd^ or 3^rd^ line nivolumab, 206 (39%) were positive for *KRAS* mutation. *KRAS* status did not influence nivolumab efficacy in terms of ORR (20% vs 17%, p = 0.39) nor DCR (47% vs 41%, p = 0.23) in patients with *KRAS*-mutant tumors when compared to *KRAS-*wild type (wt). No statistically significant differences were found in the median progression-free survival (mPFS) nor in the median overall survival (mOS) between both groups, although the 3-months PFS was significantly higher in KRAS-mutant patients (53% vs 42%, p = 0.01) (37). On the other hand, in the phase 3 study CA209-057, patients who had progressed to previous platinum-based chemotherapy (ChT), were randomized to receive either nivolumab or docetaxel. Among the 582 patients studied, *KRAS* was tested in 185 patients and 33% showed a *KRAS* mutation. When comparing the latter with *KRAS-wt* patients in the groups who received ICI, the hazard ratio (HR) for OS and PFS were 0.52 (95% confidence interval [CI]: 0.29-0.95) and 0.82 (95% CI: 0.47-1.43), respectively, in favor to *KRAS* mutant patients (38).

Regarding pembrolizumab, the clinical outcomes of two phase 3 clinical trials including *KRAS* mutant population have been reported. The KEYNOTE-042 trial (NCT0222089) evaluated pembrolizumab compared to platinum-based ChT as first-line treatment for PD-L1 positive tumors. The exploratory analysis presented by Herbst et al. showed that, out of 301 patients, 22.9% harbored KRAS mutations (9.6% were KRAS G12C) and presented higher levels of PD-L1 and tissue TMB. Both mOS and mPFS favored *KRAS* mutant tumors when treated with pembrolizumab compared to *KRAS-wt*, with a HR of 0.42 (95% CI: 0.22-0.81) and 0.51 (95% CI: 0.29-0.87), respectively (39). On the other hand, the KEYNOTE-189 trial evaluated platinum-based ChT either alone or in combination with pembrolizumab as first-line setting in advanced disease. Among 289 patients, 30.8% harbored a *KRAS* mutation and 12.8% a *KRAS G12C*. These tumors also presented higher levels of PD-L1 and higher tissue TMB. Pembrolizumab-based therapy was associated with improved clinical outcomes in terms of OS, PFS, and ORR regardless of *KRAS* status (40). Finally, results from real-world data published by Frost et al. from a multicenter, retrospective study evaluated the efficacy of first-line pembrolizumab in 119 patients with *KRAS* mutant LuAD with high PD-L1 expression (≥50%). Co-mutations in *TP53* were also evaluated, and patients with *KRAS G12C/TP53* had significantly higher ORR (100% vs 27.3%; p = 0.003) and longer mPFS (33.3 vs 2.8 months; HR, 0.18; 95% CI: 0.06-0.53; p = 0.002) than tumors with *KRAS nonG12C/TP53* mutations (41), suggesting that *KRAS G12C* present better outcomes to immune-based therapies. Furthermore, Noordhof et al. reported another retrospective study that evaluated the outcomes of first-line pembrolizumab in 595 patients with metastatic LuAD and high PD-L1 expression. *KRAS* mutations were found in 57% of the cases. Although not statistically significant, mOS with ICI was higher in *KRAS* mutant patients than in those with *KRAS-wt* (19.2 vs 16.8 months; p = 0.86) (42).

In relation to atezolizumab, efficacy results are derived from a single trial, IMpower 150 (NCT02366143), a phase 3 study with first-line ChT and bevacizumab in combination or not with atezolizumab in patients with metastatic NSCLC. An exploratory analysis evaluated atezolizumab efficacy in *KRAS* mutant population according to *STK11/KEAP1* mutation status. Among 920 evaluable patients, 24.5% harbored a *KRAS* mutation, which in 45% of the cases were co-mutated with *STK11* and/or *KEAP1*. Greater benefits in terms of OS and PFS were observed in patients harboring *KRAS* mutations in the immunotherapy-based arm, regardless of *STK11* and *KEAP1* status (43).

Finally, results on durvalumab in stage III NSCLC patients after ChT-radiotherapy are available from a retrospective study that was carried out in 134 patients from MD Anderson Hospital. Patients with driver oncogenic mutations, including *KRAS* mutations (n=26) and targetable driver mutations (n=24) in *EGFR, ALK* translocations, *ROS1* fusions, *MET exon 14 skipping, RET* fusion, and/or *BRAF*, had significantly worse mPFS compared to those without driver mutations (n=84) (8.9 months vs 26.6 months; HR 2.62 p < 0.001), particularly in cases with *KRAS* mutant tumors (mPFS 7.9 months, HR 3.34, p < 0.001), with no impact on OS based on driver mutation status (44).

## Targeting KRAS Beyond KRAS G12C Mutations

KRAS is a GTPase that, when mutated, loses the ability to turn back to the GDP-bound state and leads to a constitutively active GTP-bound state. This, in turn, activates downstream signaling pathways, including MAPK, PI3K/AKT/mTOR, and Ras-like GEF, among others, all of them responsible for cell proliferation and survival (45, 46) (**Figure 1**). In addition, the heterogeneity of KRAS mutations results in a variety of different diseases, which hinders the finding of a unique common therapy to address all of them. Thus, while KRAS G12C specific inhibitors have proven efficacy against their target, many other therapeutic strategies are currently under development for those *KRAS* mutant tumors with no druggable genetic alteration (47). Clinical trials addressed to *KRAS* mutant NSCLC non specific for KRAS G12C are listed in **Table 1**.

**Table 1:** Clinical trials of drugs targeting KRAS.

Therapeutic Family	Clinical Trial	Phase	Drug	Indication	Results
Pan-RASinh	NCT03114319	1	TNO155 (SHP2i) alone or with nazartinib (EGFRi)	EGFR/KRAS NSCLC, esophageal SCC, H/N SCC, Melanoma	N/A
	NCT03634982	1	RMC-4630 (SHP2i)	All solid tumors	N/A
	NCT04045496	1	JAB-3312 (SHP2i)	All solid tumors	N/A
Pan-RASinh +downstream inh	NCT04111458	1	BI 1701963 (SOS1i) + trametinib (MEKi)	KRAS NSCLC	N/A
	NCT04916236	1	RMC-4630 (SHP2i) + LY3214996 (ERK1/2i)	KRAS tumors	N/A
Pan- RASinh + IT	NCT04000529	1b	TNO155 (SHP2i) + ribociclib (CDK4/6i) or spartalizumab (PD1i)	KRAS NSCLC	N/A
Downstream inh	NCT03681483	1	RO5126766 (RAF/MEKi)	KRAS NSCLC	N/A
Downstream inh combination	NCT02857270	1	LY3214996 (ERK1/2i) alone or + other drugs	All solid tumors	N/A
	NCT03284502	1b	HM95573 (RAFi) + cobimetinib (MEKi)or cetuximab (EGFRi)	All solid tumors	N/A
	NCT03170206	1/2	palbociclib (CDK 4/6i) + binimetinib (MEKi)	KRAS NSCLC	N/A
	NCT04620330	2	VS-6766 (RAF/MEKi) + defactinib (FAKi)	G12V or other KRAS NSCLC	N/A
	NCT02974725	1	LXH254 (RAFi) + LTT462 (ERK1/2i) or trametinib (MEKi) or ribociclib (CDK4/6i)	KRAS and BRAF tumors	N/A
Downstream inh + Upstream inh	NCT01229150	2	selumetinib (MEKi) + erlotinib (EGFRi) vs selumetinib	NSCLC	ORR 10% vs 0% OS 21.8 vs 10.5 months
	NCT02230553	1/2	trametinitb (MEKi) + lapatinib (Erbb1-2i)	KRAS NSCLC	N/A
	NCT03704688	1/2	trametinib (MEKi) + poniotinib (VEGFi)	KRAS NSCLC	N/A
	NCT04967079	1	trametinib (MEKi) + anlotinib (panRTKi)	KRAS NSCLC	N/A
	NCT01859026	1/2.	MEK162 (MEKi) + erlotinib	KRAS or EGFR tumors	N/A
	NCT04965818	1b/2	futibatinib (FGFRi) + binimetinib (MEKi)	KRAS tumors	N/A
Downstream inh + autophagy inh	NCT04735068	2	binimetinib (MEKi) + hydroxychloroquine	KRAS NSCLC	N/A
	NCT04892017	1	DCC-3116 (ULK 1/2i) + trametinib (MEKi)	RAS-RAF mutant all solid tumors	N/A
Autophagy inh	NCT03095612	1/2	selinezor (XPO1i) + docetaxel	KRAS NSCLC	N/A
Downstream inh + IT	NCT02779751	1b	pembrolizumab (PD1i) + abemaciclib (CDKi)	KRAS non squamous NSCLC, sq-NSCLC and Luminal-like breast cancer	N/A
	NCT02779751	1b	pembrolizumab (PDL1i) + abemaciclib (CDKi)	KRAS non squamous NSCLC, sq-NSCLC and Luminal-like breast cancer	N/A
	NCT03299088	1b	pembrolizumab (PD1i) + trametinib (MEKi)	KRAS NSCLC	N/A
Downstream inh + ChT	NCT03990077	1	HL-085 (MEKi) + docetaxel	KRAS NSCLC	N/A
mRNA vaccine	NCT03948763	1	V941 (mRNA vaccine)	All solid tumors	N/A
Metabolic modifier	NCT03808558	2	TVB-2640 (FASNi)	KRAS NSCLC	N/A

Inh/i, inhibitors; IT, immunotherapy; ChT, chemotherapy; NSCLC, non-small cell lung cancer; SCC, squamous cell carcinoma; ORR, overall response rate; N/A, non-assessed.

Before the advent of KRAS G12C inhibitors, several strategies were tested to target the broad spectrum of KRAS mutant tumors, but most of them failed. Historically, the majority of these strategies focused on downstream effectors. For instance, the MEK inhibitor selumetinib combined with docetaxel showed good responses in early trials but failed to improve survival in a randomized phase 3 trial (48, 49). The cyclin-dependent kinase (CDK4/6) inhibitor abemaciclib was also tested in a randomized phase 3 trial against erlotinib but could not achieve its OS primary endpoint (50). Combining downstream effector inhibitors targeting MEK/PI3K demonstrated moderate responses but unacceptable toxicity profiles (51). Another therapeutic strategy focusing on the RAS family was the interruption of its anchoring to the cell membrane by inhibiting the post-translational farnesylation (e.g. tipifarnib). Unfortunately, despite a seemingly effective strategy in early phase trials, phase 2 and 3 trials assessing the effectivity in *KRAS* mutant tumors could not meet the expected outcomes (52). Hence, ChT remained the main treatment for these tumors for a long time, albeit with limited success.

Since *KRAS* mutant tumors form a heterogenous disease, nowadays, investigational efforts are focused on a subset of targeted therapies that can be further classified in different families according to their mechanism of action. Furthermore, to achieve better outcomes, the different families can be combined with themselves, with KRAS G12C inhibitors, or with immunotherapy. We will subsequently develop further each one of these potential options:

i) KRAS Mutation-Specific Inhibitors
Besides KRAS G12C inhibitors, no other point mutations of *KRAS* have been successfully targeted in human trials yet. However, the artificial cyclic peptide KS-58 enhanced anti-cancer activity *in vitro* and *in vivo* in *KRAS G12D* mutant tumors by blocking intracellular Ras-effector protein interactions (53). Also, the new molecule MRTX1133 has shown promising results, binding to G12D in lung and pancreatic tumor models (54).
ii) Pan-KRAS Inhibitors
SOS1 is a guanine exchange factor for KRAS promoting the phosphorylation of GDP to GTP by binding to its catalytic site. Moreover, SOS1 can bind to the allosteric site of KRAS that potentiates its GEF function, increasing its positive feedback regulation (54). SOS1 inhibition has demonstrated a depletion effect on tumors that depend on KRAS activation. Recently, a new potent and selective SOS1 inhibitor, BI-3406, has shown *in vitro* and *in vivo* antitumor activity (55). The drug decreased GTP-loaded KRAS and attenuated feedback reactivation by MEK inhibitors, suggesting that this combination may be a promising treatment option. In fact, that specific combination was tested *in vitro* in cell lines resistant to KRAS G12C inhibitors with satisfactory results and is currently ongoing phase 1 trials (56). SHP2 is a protein tyrosine phosphatase existing either bound to the cytoplasmatic portion of an activated RTK or as a component of the RAS activating complex (57). SHP2 allosteric inhibitors, such as TNO155 and RMC-4630, have shown activity on ERK inhibition; ongoing trials combining both treatments are currently assessing their potential to overcome drug resistance to RTK/RAS/MAPK inhibitors (58, 59).
iii) Downstream KRAS Inhibitors
The immediate effect of activated RAS is the interaction between the RAS switched region with the RAS binding domain (RBD) conserved in multiple proteins of multiple signaling pathways. A RAS mimetic small molecule, rigosertib, targets these RBD by interrupting RAF, RALGDS, and PI3Ks signaling cascades (60). So far, RAF and MEK inhibitors alone or in combination with RTK inhibitors have failed to prove effective in *KRAS* mutant tumors. However, a novel RAF/MEK potent inhibitor showed a 60% tumor reduction in *RAS-RAF* mutated tumors and is being tested in combination with a FAK inhibitor in *KRAS* mutant LuAD (61). ERK 1/2 inhibitors are yet to be approved but are expected to directly suppress the MAPK pathway’s effector node. When administered alone, only achieved disease stabilization and the phase I trial testing LT462 (NCT02711345) was terminated earlier. ERK inhibitors could further be combined with drugs targeting upstream nodes in the MAPK pathway to reduce the incidence of acquired resistance. Besides, the PI3K/mTOR/AKT pathway is a downstream pathway activated by the RAS family. In this early phase clinical trial (NCT00933777), the combination of sorafenib (a multi-TKI) with everolimus (mTOR inhibitor) did not achieve any partial response in NSCLC patients harboring *KRAS* mutation, assessed by CT-scans (62). However, combinations of MEK and PI3K/mTOR/AKT inhibitors have demonstrated better efficacy in *KRAS* mutant LuADthan either one alone, but their potential toxicity has to be addressed by different treatment schemes (e.g. intermittent dosage) (63, 64).
iv) Upstream KRAS Inhibitors
*KRAS*-mutant tumors are still sensitive to extracellular growth factors and not completely independent of growth factor receptors (ERBB proteins), since both have a role in tumorigenesis (65). Their activity could be tackled by blocking upstream effectors and the combination of pan-Erbb family inhibitors with KRAS pathway inhibitors could increase the efficacy as well as contribute to overcome the drug resistance enhancing the outcomes observed with inhibiting each target alone, although at cost of increased toxicities. Two preclinical studies further support the idea that mutant *KRAS* demands activation of ERBB receptors to facilitate lung tumorigenesis (65, 66). One approach was focused on combining KRAS inhibition with epidermal growth factor receptor (EGFR) TKIs, as the EGFR signaling pathway is often activated in tumor cells to bypass KRAS inhibition. However, past clinical trials combining MEK inhibitor, selumetinib with EGFR inhibitor, Erlotinib were largely unsuccessful with an ORR of 10% (95% CI 2.1 to 26.3%) in *KRAS* mutant patients (67).
v) Cellular Metabolism and Autophagy
*KRAS* mutant tumors present a high glucose metabolism, so that, multiple glycolytic genes are upregulated and its suppression could prevent tumor growth (68). Autophagy is a strategy to overcome starvation in healthy cells and it has been observed to be increased in many cancer types. Autophagy prevents cells from undergoing programmed cell death. Moreover, it has been shown that the RAF/MEK/ERK cascade leads to autophagy *via* STK11/AMPkinase-activated (AMPK) protein that activates the autophagy kinase 1 signaling axis (69). Combined inhibition of autophagy and MAPK signaling is nowadays being studied in phase 1 and 2 trials (NCT04892017) (70).

## Conclusions

The high frequency of *KRAS* mutations in cancer justifies the multiple efforts invested in developing novel therapeutic strategies targeting KRAS. A deeper understanding of the cancer biology and immune system interactions that fuel carcinogenesis in *KRAS* mutant tumors is essential for developing new drugs and improving disease prognosis. Besides KRAS G12C specific inhibitors, several other drugs targeting KRAS directly or indirectly are being investigated. In addition, the list of actionable *KRAS* mutations in lung cancer will likely increase in the upcoming years.

Current immunotherapies seem to be effective for subset of *KRAS* mutant tumors, due in part, by the influence of smoking related nature of *KRAS G12C* mutations. The presence of co-mutations such as *STK11* or *KEAP1* shape the tumor immune microenvironment and might has an impact on treatment efficacy. Incorporating these genetic alterations in diagnostic panels as predictive markers represent a useful strategy for therapeutic decisions, including immunotherapy-based regimens.

Finally, the genomic complexity of *KRAS* mutant tumors will ultimately require tailored application of therapeutic approaches and upcoming data from clinical trials will contribute to provide the most promising strategies.

## Author Contributions

Conceptualization, EC, MC, and MS. Writing—original draft preparation, MC, EC, and MS. Writing—review and editing, MS-C, CH, LN, and AE. Supervision, MS, MC, EC, and MS-C. All authors have read and agreed to the published version of the manuscript.

## Funding

This research received no external funding. MS is supported by a Juan-Rodés contract from the Instituto de Salud Carlos III (JR20/00015).

## Conflict of Interest

MC reports advisory/consultancy: Roche, Bristol-Myers Squibb, AstraZeneca; Travel/expenses: Pfizer, Roche. MSC reports a sponsored research agreement with Merck Serono Pharmaceuticals. CH reports research grants from Merck, and speaker’s bureau from MSD, Lilly and Ipsen. AE reports honoraris from Roche, MSD, AstraZeneca and Pharmamar. Travel/expenses: Roche, MSD, AstraZeneca, Lilly, Pfizer, Pharmamar. EC reports advisory/consultancy: AstraZeneca, Boehringer Ingelheim, Bristol Myers Squibb, MSD, Novartis, Roche, Takeda. Speaker bureau: AstraZeneca, Boehringer Ingelheim, Bristol-Myers Squibb. MSD, Novartis, Pfizer, Roche, Takeda, Amgen. Travel/accommodation/expenses: Bristol Myers Squibb, Pfizer, Roche, Takeda. MS reports a sponsored research agreement with Merck Serono Pharmaceuticals. Advisory/consultancy: Roche, Bristol-Myers Squibb, AstraZeneca, Takeda; Travel/expenses: Pfizer, Roche.

The remaining author declares that the research was conducted in the absence of any commercial or financial relationships that could be construed as a potential conflict of interest.

## Publisher’s Note

All claims expressed in this article are solely those of the authors and do not necessarily represent those of their affiliated organizations, or those of the publisher, the editors and the reviewers. Any product that may be evaluated in this article, or claim that may be made by its manufacturer, is not guaranteed or endorsed by the publisher.
